# Integrated Genomic and Transcriptomic Analyses of Diffuse Large B-Cell Lymphoma With Multiple Abnormal Immunologic Markers

**DOI:** 10.3389/fonc.2022.790720

**Published:** 2022-02-14

**Authors:** Lingshuang Sheng, Di Fu, Yiwen Cao, Yujia Huo, Shuo Wang, Rong Shen, Pengpeng Xu, Shu Cheng, Li Wang, Weili Zhao

**Affiliations:** Shanghai Institute of Hematology, State Key Laboratory of Medical Genomics, National Research Center for Translational Medicine at Shanghai, Ruijin Hospital, Shanghai Jiao Tong University School of Medicine, Shanghai, China

**Keywords:** immune abnormalities, omic analyses, oxidative phosphorylation, cell cycle, immune response, DLBCL—diffuse large B-cell lymphoma

## Abstract

**Background:**

Diffuse large B-cell lymphoma (DLBCL) is a highly aggressive subtype of lymphoma and related to autoimmune diseases (AIDs). Primary B-cell receptor-mediated AIDs are associated with poor clinical outcome of DLBCL. To further determine the role of immunological alterations on disease progression, our study integrated genomic and transcriptomic analyses on DLBCL with multiple abnormal immunologic markers.

**Methods:**

The clinical data of 1,792 patients with newly diagnosed DLBCL were collected, with DNA- and RNA-sequencing conducted for 164 and 127 patients, respectively. Frequent gene mutations and the involved dysregulated pathways, along with gene expression pattern and tumor microenvironment alternations, were analyzed and compared based on the immune status of the patients.

**Results:**

DLBCL with multiple abnormal immunologic markers demonstrated a variety of characteristics including elevated serum lactic dehydrogenase level, inferior prognosis, and dysregulated cell cycle and immune response, as well as activated oxidative phosphorylation pathway and increased Th1/Th2 and Th17/Treg ratios, which were highly similar as those that occur in AIDs.

**Conclusions:**

We piloted the description of the clinical and genetic features of DLBCL with multiple abnormal immunologic markers, illustrated possible mechanisms of disease progression, and provided a clinical rationale of mechanism-based targeted therapy in this subset of DLBCL.

## Introduction

Diffuse large B-cell lymphoma (DLBCL) is the most common type of non-Hodgkin lymphoma (NHL) worldwide and represents a group of heterogeneous diseases with variable clinical features, genetic characteristics, treatment response, and disease outcome (1). With durable remission achieved in 50%–60% of the patients upon immunochemotherapy with rituximab, cyclophosphamide, doxorubicin, vincristine, and prednisone (R-CHOP), the prognosis of DLBCL patients is impacted by multiple factors, including cell-of-origin (COO), BCL-2/MYC double expression (DEL), and double hit lymphoma (DHL). Non-germinal center B-cell-like (non-GCB), DEL, and DHL subtypes of DLBCL have worse clinical outcome (2). Epidemiological studies indicated a 5%–20% increased risk in DLBCL among patients with autoimmune diseases (AIDs) (3, 4). Although the underlying mechanism remains unclear, current consensus suggests that chronic inflammation and antigen stimulation of AIDs may lead to lymphoma pathogenesis. Immune response is defined as any immune system process that functions in calibrated responses of an organism to a potential internal or external threat according to the Gene Ontology (GO) database, which is activated in various AIDs (5–7) and results in enhanced chronic immune activities and increased disease severity. Moreover, B-cell receptor (BCR)-mediated AIDs, including rheumatoid arthritis (RA), systemic lupus erythematosus (SLE), and Sjögren’s syndrome (SS), often result in disease progression due to uncontrolled proliferation and transformation of malignant B cells (4).

Tumor cell metabolism is accelerated *via* glycolysis, so as to better support malignant cell growth and metastasis (8). However, recent studies have reported an alternative metabolic pathway *via* oxidative phosphorylation (OxPhos) and ribosome, including DLBCL (9, 10). DLBCL can be divided into three subtypes: OxPhos-DLBCL, BCR-DLBCL, and host response (HR)-DLBCL. OxPhos-DLBCL is characterized by increased expression of proteasomal subunits and molecules, which regulate mitochondrial membrane potential and apoptosis and could be sensitive to proteosome blockade or inhibition of B-cell lymphoma-2 (BCL-2) family (11). For the biological process cell cycle, *CCND3* mutation frequently occurs in DLBCL with multiple abnormal immunologic markers and is involved in uncontrolled cell cycle (12), the activation of which contributes to DLBCL progression (13).

Our previous study showed that DLBCL with multiple (three or greater) abnormal immunologic markers is significantly associated with shorter 3-year progression-free survival (PFS) and overall survival (OS) than those without multiple abnormal immunologic marker (14). Here, we collected the clinical data of 1,792 patients with newly diagnosed DLBCL and conducted multi-omics study to characterize DLBCL with multiple abnormal immunologic markers.

To our knowledge, this was the first study on the association of genomic, transcriptomic, and tumor microenvironment alterations with abnormal immune status in DLBCL. DLBCL with multiple abnormal immunologic markers was featured by dysregulated cell cycle and immune response and activated OxPhos pathway. OxPhos may act as a crucial factor during this process that functions *via* 1) promoting B-cell clonal expansion and positive selection in germinal centers (GCs), 2) regulating T-cell subsets, and 3) providing sufficient energy for lymphoma cells.

## Materials and Methods

### Patients

**Figure 1:**
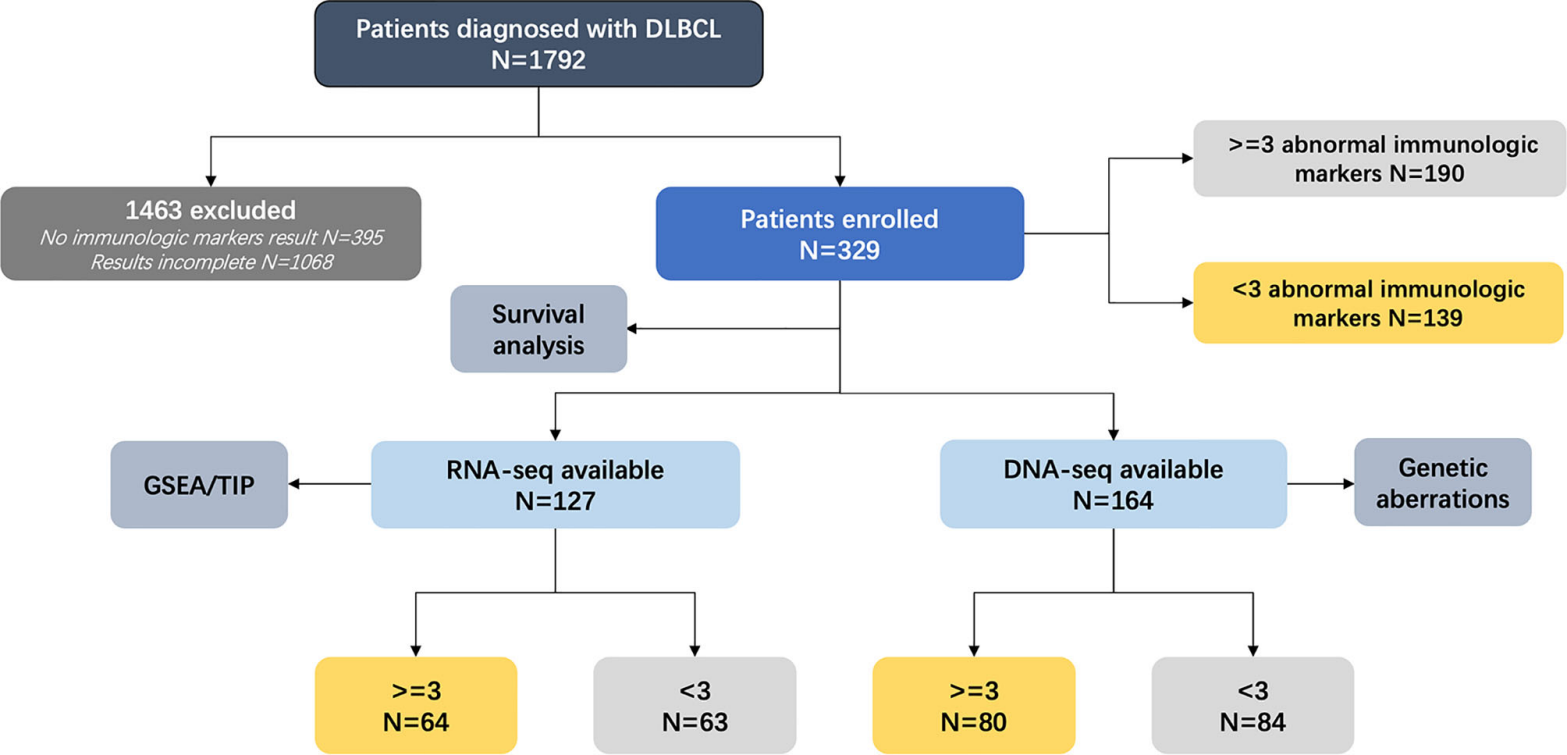
Analytical methodology of enrolled patients. DLBCL, diffuse large B-cell lymphoma; DNA-seq, DNA-sequencing; RNA-seq, RNA-sequencing; GSEA, gene set enrichment analysis; TIP, tracking tumor immunophenotyping.

The flowchart of the patients enrolled in our study is described in **Figure 1**. The clinical data of 1,792 patients with newly diagnosed DLBCL from January 2000 to January 2020 were collected. With 1,463 patients excluded due to missing or incomplete immunologic marker data, 329 patients were divided into two cohorts according to the number of abnormal immunologic markers: 190 patients with multiple (three or greater) (14) abnormal immunologic markers as the abnormal group and 139 patients with fewer than three abnormalities as the normal group based on our previous study (14). Immunologic markers include serum immunoglobulins G (IgG), IgM, IgA, and IgE; circulation immunity compound (CIC); rheumatoid factors (RF); anti-dsDNA; anti-Sjögren’s syndrome-related antigen (anti-SSA); antinuclear antibodies (ANA); anti-streptolysin “O” (ASO); and complements (C3 and C4). Except for C3 and C4 whose decrease is referred to as abnormal, the increase of other biomarkers is referred to as abnormal. Among 329 patients, DNA- and RNA-sequencing were performed on 164 (80 in the abnormal group and 84 in the normal group) and 127 patients (64 in the abnormal group and 63 in the normal group), respectively. Survival analysis was conducted for all enrolled patients. Histological diagnosis was established based on the revised 2017 World Health Organization (WHO) classification (fourth edition) (15). Among all patients, 97.57% (321/329) received standard R-CHOP-based immunochemotherapy. Apart from R-CHOP, first-line therapy included standard R-DA-EDOCH (rituximab, dose-adjusted etoposide, dexamethasone, vincristine, cyclophosphamide, and doxorubicin, 2/329), R-COP (5/329), and other non-anthracycline-containing regimens (IR2, ibrutinib, rituximab, and lenalidomide, 1/329). All patients were evaluated by PET/CT after 3–8 cycles of first-line therapy according to the Lugano 2014 classification (16).

A variety of clinical data including gender, age, number of extranodal involvement, serum lactic dehydrogenase (LDH), performance status (Eastern Cooperative Oncology Group, ECOG), Ann Arbor stage, International Prognostic Index (IPI) score, response to first-line therapy, PFS, and OS were collected. As for pathological subtypes, GCB or non-GCB subgroups were determined using the Hans classification (17), with 30% cutoff values for CD10, BCL-6, and MUM-1. For BCL-2/MYC DEL, the cutoff values for BCL-2 and MYC were 50% and 40%, respectively (18, 19). The COO algorithm categorizing DLBCL into GCB, activated B-cell-like (ABC), and unclassified subtype (UN) was established using RNA-sequencing data (20). Genetic subtypes were classified by leveraging LymphGen algorithm (21). IgM, IgA, IgE, CIC, C3, C4, RF, anti-SSA, and ASO were assessed by turbidimetric inhibition immunoassay (Beckman Coulter, CA, USA). Anti-dsDNA and ANA were assessed by ELISA (Inova, CA, USA). The study was approved by Shanghai Ruijin Hospital Ethics Board and informed consent was obtained from all patients in accordance with the Declaration of Helsinki.

### DNA-Sequencing

Targeted sequencing was performed on frozen tumor tissue samples or qualified formalin-fixed paraffin-embedded tumor tissue samples of DLBCL. PCR primers were designed by Primer 5.0 software. Multiplexed libraries of tagged amplicons from tumor samples were generated by Shanghai Righton Bio-Pharmaceutical Multiplex-PCR Amplification System. Deep sequencing was performed with Illumina HiSeq 4000 platform protocols. Pathways and related genes (based on the GO database) were presented as follows: histone/DNA methylation (*TET2*, *KMT2C*, *KMT2D*, *HIST1H1C*, *HIST1H1E*), histone acetylation (*EP300*, *CREBBP*), immune response (*CD58*, *B2M*, *CIITA*, *IRF4*, *NOTCH2*), cell cycle (*ATM*, *EP300*, *CCND3*, *MYC*), Wnt signaling pathway (*DDX3X*, *FOXO1*, *GNA13*, *TBL1XR1*), BCR/NFκB signaling pathway (*CARD11*, *CD79B*, *MYD88*, *PIM1*, *PTPN6*, *NFKBIE*), TNFR/NFκB signaling pathway (*TNFAIP3*, *TNFRSF14*, *CD70*, *TMSB4X*, *NFKBIE*, *PTPN6*), JAK–STAT pathway (*SCOS1*, *STAT3*, *STAT6*, *IRF8*, *NOTCH1*, *BCL6*), and PI3K–AKT pathway (*MTOR*, *TSC2*, *MYC*, *SGK1*).

### RNA-Sequencing

Total RNA was extracted from frozen tumor tissue samples by TRIzol and RNeasy Mini Kit (QIAGEN, Hilden, Germany), and the integrity of total RNA was evaluated by RNA 6000 Nano Kit on Agilent 2100 Bioanalyzer. Read pairs were aligned to Refseq hg19 with Burrows-Wheeler Aligner version 0.7.13-r1126. Transcript counts table files were generated *via* HTSeq (22). Potential false-positive results were excluded *via* visual inspection. Bioinformatic analyses were performed through R 3.5.1, with R package “sva” for batch effect removal. Raw reads were normalized, and differentially expressed genes were obtained with R package “limma” (v3.38.3).

### Gene Set Enrichment Analysis

Gene set enrichment analysis (GSEA) was conducted with GSEA v4.1.0 software and Molecular Signature Database (MSigDB) v7.4 (23, 24). The metric for ranking genes was Signal2Noise by default. Phenotypes that contained at least seven samples were labeled permutation type. Based on the GSEA team recommendation (http://www.broadinstitute.org/gsea), the statistical significance of enrichment score was assessed with permutation being set up at 1,000. Enriched pathways were considered statistically significant with *P*-value under 0.05 and false discovery rate under 0.25.

### Tracking Tumor Immunophenotyping

The state of antitumor immunity was analyzed and visualized with the tracking tumor immunophenotyping (TIP) (http://biocc.hrbmu.edu.cn/TIP) method (25) that contains 178 signature genes and 23 signature gene sets involved in cancer-immunity cycle and could thus grade the recruitment of specific T-cell subsets from published studies (26). With the gene expression data collected, activity scores of the gene sets were calculated separately, based on their stimulatory or inhibitory role in antitumor immune response. The final score of each signature gene set of each individual sample was calculated by examining the difference between the normalized scores of stimulatory and inhibitory gene sets.

### Statistical Analysis

Baseline characteristics of patients were ascertained using Pearson’s *χ* ^2^ test or Fisher’s exact test. The difference of immunity activity scores and normalized gene expression in the two groups were analyzed using the Mann–Whitney *U* test. PFS was defined as the time period between initial diagnosis and disease progression, relapse, or last follow-up. OS was defined as the time period between initial diagnosis and date of death or last follow-up. Survival analyses were estimated using the Kaplan–Meier method and compared by the log-rank test. Univariate hazard estimates were generated with unadjusted Cox proportional hazards models. Statistical significance was defined as *P <*0.05. All *P*-values in this manuscript were reported without mathematical correction. The above statistical analyses were performed by Statistical Package for the Social Sciences (SPSS) 26.0 software (SPSS Inc., Chicago, IL, USA).

## Results

### Frequent Gene Mutations and Involved Dysregulated Pathways

**Figure 2:**
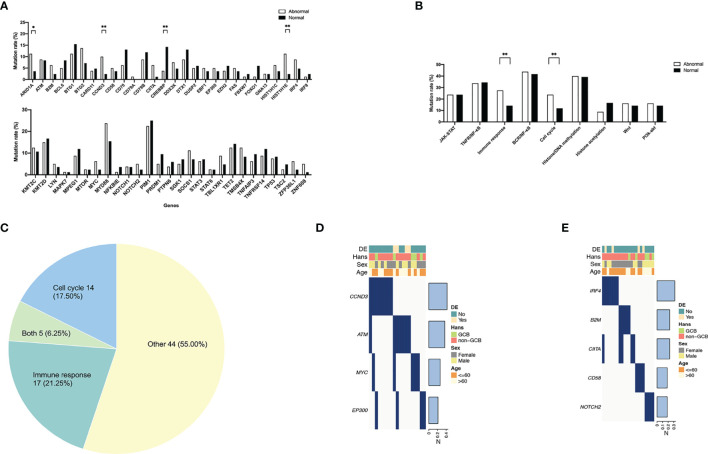
Frequent gene mutations and involved pathways in diffuse large B-cell lymphoma according to different immune status. **(A)** Gene mutations of the abnormal (*n* = 80) and the normal (*n* = 84) group. **(B)** Dysregulated pathways of the abnormal and the normal group. *P*-values comparing between different mutation rates in the two groups are marked as “**” if less than 0.05 and “*” if less than 0.1 as shown above. **(C)** Molecular subtypes in the abnormal group. “Both” represents patients with both molecular subtypes of cell cycle and immune response genetic characteristics. **(D)** Gene mutation distribution of patients with cell cycle subtype (*n* = 21). **(E)** Gene mutation distribution of patients with immune response subtype (*n* = 21).

As shown in **Figure 2A**, the mutation rates of *CCND3* (8/80 vs. 2/84, *P* = 0.042) and *HIST1H1E* (9/80 vs. 2/84, *P* = 0.023) were significantly higher in the abnormal group than in the normal group. More importantly, when categorizing genes into oncogenic signaling pathways, i.e., JAK–STAT, BCR/NFκB, TNFR/NFκB, Wnt, and PI3K–AKT, as well as biological processes, i.e., immune response, cell cycle, histone/DNA methylation, and histone acetylation, the mutation rates of cell cycle (19/80 vs. 10/84, *P* = 0.047) and immune response (22/80 vs. 12/84, *P* = 0.037) were significantly activated in the abnormal group, as compared with the normal group (**Figure 2B**). Patients with gene mutations of cell cycle and immune response represented 19 (23.75%) and 22 (27.50%) of 80 patients, respectively (**Figure 2C**). Meanwhile, 5 of these patients demonstrated both cell cycle and immune response subtypes. The rest of the cases were defined as other subtype, and they marked up more than a half (44/80, 55.00%) of all patients. The distribution of gene mutations of the cell cycle and immune response subtypes is shown in **Figures 2D, E**. The most frequent mutated genes were *CCND3* (8/19, 42.11%) in the cell cycle subtype and *IRF4* (7/22, 31.82%) in the immune response subtype, respectively.

### Clinical Outcome

**Figure 3:**
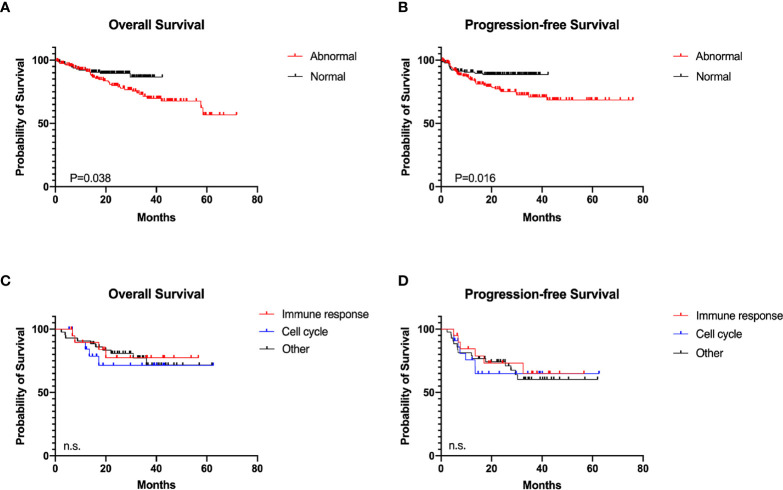
Survival analyses in diffuse large B-cell lymphoma according to different immune status. **(A)** Overall survival (OS) of the abnormal (n = 190) and the normal (n = 139) group. **(B)** Progression-free survival (PFS) of the abnormal and the normal group. **(C)** OS of the molecular subtypes (21 cell cycle subtype, 21 immune response subtype, and 44 others) in the abnormal group. **(D)** PFS of molecular subtypes in the abnormal group.

The clinical and pathological features of 190 patients in the abnormal group and 139 in the normal group were analyzed (**Table 1**). Serum LDH level of the abnormal group was significantly higher than that of the normal group (104/190 vs. 59/139, *P* = 0.028). The number of EZB genetic subtype DLBCL patients was significantly increased in the normal group (5/44 vs. 0/52, *P* = 0.013), which might indicate improved prognosis (21). No significant difference was observed between the two groups in DEL and COO. Among all patients, 97.37% (185/190) of the abnormal group and 96.40% (134/139) of the normal group received standard R-CHOP-based immunochemotherapy, and 96.84% (184/190) of the abnormal group and all patients of the normal group received standard anthracycline-containing regimen. The overall response rate (ORR) of first-line therapy among patients was 75.26% (143/190) in the abnormal group and 71.94% (100/139) in the normal group, respectively. Survival analyses were further conducted, with an *ad-hoc* analysis performed for 80 patients from three molecular subtypes (cell cycle, immune response, and other subtype) in the abnormal group. The 2-year PFS and OS of the abnormal group were 75.17% and 79.87%, significantly shorter than those of the normal group (88.58%, *P* = 0.016, and 89.48%, *P* = 0.038, **Figures 3A, B**). Hazard ratio and 95% confidence interval (95% CI) of PFS and OS were 2.08 (95% CI 1.20 to 3.62) and 1.86 (95% CI 1.07 to 3.25), respectively. No statistical significance of prognosis was observed among the three molecular subtypes. The 2-year PFS and OS of the cell cycle, immune response, and other subtypes were 71.45% vs. 77.42% vs. 80.78% and 64.94% vs. 73.19% vs. 74.19%, respectively (**Figures 3C, D**).

**Table 1:** Clinical characteristics of the patients with DLBCL.

		Abnormal (*n* = 190)	Normal (*n* = 139)	*P*-value
**Age**	>60	96 (50.5%)	69 (49.6%)	0.874
	≤60	94 (49.5%)	70 (50.4%)	
**Gender**	Male	86 (45.3%)	73 (52.5%)	0.193
	Female	104 (54.7%)	66 (47.5%)	
**B symptoms**	No	141 (74.2%)	111 (79.9%)	0.232
	Yes	49 (25.8%)	28 (20.1%)	
**ECOG score**	0–1	158 (83.2%)	117 (84.2%)	0.806
	≥2	32 (16.8%)	22 (15.8%)	
**Ann Arbor stage**	I–II	83 (43.7%)	71 (51.1%)	0.184
	III–IV	107 (56.3%)	68 (48.9%)	
**LDH level**	Normal	86 (45.3%)	80 (57.6%)	0.028
	Elevated	104 (54.7%)	59 (42.4%)	
**Extranodal sites**	0–1	135 (71.1%)	89 (64.0%)	0.177
	≥2	55 (28.9%)	50 (36.0%)	
**IPI score**	0–2	103 (54.2%)	86 (61.9%)	0.165
	3–5	87 (45.8%)	53 (38.1%)	
**Pathological subtypes**	DLBCL-NOS	184 (96.8%)	134 (96.4%)	0.827
	EBV^+^ DLBCL	5 (2.6%)	3 (2.2%)	0.783
	PCDLBCL-LT	1 (0.5%)	2 (1.4%)	0.390
**Hans classification**	GCB	59 (31.1%)	57 (41.0%)	0.062
	Non-GCB	131 (68.9%)	82 (59.0%)	
**DEL**	No	162 (85.3%)	125 (89.9%)	0.210
	Yes	28 (14.7%)	14 (10.1%)	
**Cell-of-origin (COO)**	GCB	20/64 (31.3%)	20/69 (29.0%)	0.776
	ABC	24/64 (37.5%)	37/69 (53.6%)	0.062
	UN	20/64 (31.3%)	12/69 (17.4%)	0.061
**Genetic subtypes**	A53	4/52 (7.7%)	1/44 (2.3%)	0.234
	BN2	4/52 (7.7%)	4/44 (9.1%)	0.805
	EZB	0/52 (0.0%)	5/44 (11.4%)	0.013
	MCD	3/52 (5.8%)	4/44 (9.1%)	0.533
	ST2	2/52 (3.9%)	0/44 (0.0%)	0.189
	Other	39/52 (75.0%)	30/44 (68.2%)	0.460
**Treatment response**	CR/PR	143 (75.3%)	100 (71.9%)	0.498
	SD/PD	47 (24.7%)	39 (28.1%)	

### Gene Expression Pattern

**Figure 4:**
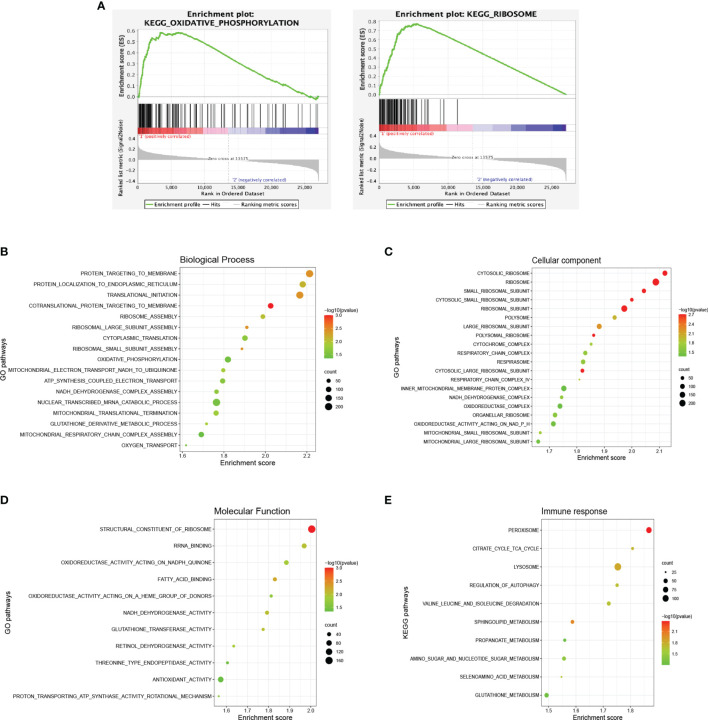
Dysregulated signaling pathways in diffuse large B-cell lymphoma according to different immune status. **(A)** Enrichment plots of oxidative phosphorylation and ribosome of the abnormal (*n* = 64) and the normal (*n* = 63) group according to the Kyoto Encyclopedia of Genes and Genomes (KEGG) database. **(B)** Enriched biological processes of the abnormal and the normal group based on the Gene Ontology (GO) database. **(C)** Enriched cellular components of the abnormal and the normal group based on the GO database. **(D)** Enriched molecular functions of the abnormal and the normal group based on the GO database. **(E)** Enriched pathways of the immune response subtype and others based on the KEGG database. The color of the points indicates −log (*P*-value) of dysregulated pathways in the two groups. The size of the points indicates the number of genes included in each gene set.

GSEA was conducted for gene enrichment based on the Kyoto Encyclopedia of Genes and Genomes (KEGG) and Gene Ontology (GO) databases using RNA-sequencing data. OxPhos (*P* = 0.044) and ribosome (*P* < 0.001) pathways were significantly upregulated according to the KEGG database in the abnormal group, as compared with the normal group (**Figure 4A**). GO database analysis suggested that OxPhos and ribosome-associated biological processes, e.g., adenosine triphosphate (ATP) synthesis mitochondria activities and nuclear transcribed mRNA catabolic process, were enriched in the abnormal group (**Figure 4B**). Cellular components including mitochondrial component complexes, OxPhos-related enzymes, and various ribosomal subunits (**Figure 4C**), along with activation of molecular functions during OxPhos and ribosome, were increased in the abnormal group as well (**Figure 4D**).

Regarding the gene expression pattern of abnormal molecular subtypes, 11 patients with cell cycle, 13 with immune response, and 30 with others were analyzed. The immune response subtype demonstrated a significantly enhanced metabolic status based on the KEGG database (**Figure 4E**). Upregulated genes were enriched mainly in various metabolic pathways, including tricarboxylic acid cycle (TCA), valine leucine and isoleucine degradation, and metabolism of propanoate, amino sugar and nucleotide sugar, selenoamino acid, and glutathione. Meanwhile, lysosome, regulation of autophagy, and sphingolipid metabolism were also activated, which might contribute to tumor angiogenesis and AIDs like amyotrophic lateral sclerosis (ALS).

We also analyzed the gene expression pattern of patients with *CCND3* mutation of the cell cycle subtype and *IRF4* mutation of the immune response subtype. Interestingly, *CCND3* mutations were related to alternation in DNA replication and cell cycle pathway (**Supplementary Figure S1A**), while *IRF4* mutations were related to alternation in mitochondrial biological processes (**Supplementary Figure S1B**). These findings further confirmed that the major mutations of the abnormal group were biologically functional.

### Tumor Microenvironment

**Figure 5:**
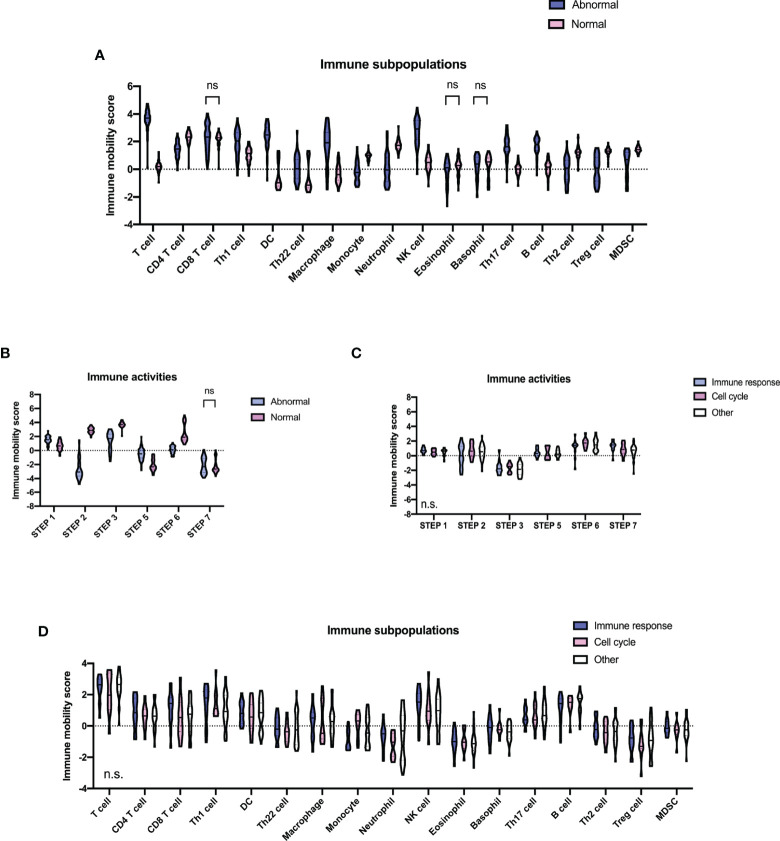
Tumor microenvironment in diffuse large B-cell lymphoma according to different immune status. **(A)** Immune subpopulations of the abnormal (*n* = 64) and the normal (*n* = 63) group. **(B)** Immune activities of the abnormal and the normal group. *P*-values comparing between different immune mobility scores of the two groups are marked as “ns” if no less than 0.1 and being unmarked if less than 0.01. **(C)** Immune activities of molecular subtypes (11 cell cycle subtype, 13 immune response subtype, and 30 others) of the abnormal group. **(D)** Immune subpopulations of molecular subtypes of the abnormal group. *P*-values comparing against different immune mobility scores among the three subtypes are shown in the lower left part of each plot. Step 1, release of cancer antigens; step 2, cancer antigen presentation; step 3, priming and activation; step 4, trafficking of immune cells to tumors; step 5, infiltration of immune cells into tumors; step 6, recognition of cancer cells by T cells; step 7, killing of cancer cells.

TIP analyses were conducted using RNA-sequencing data. Compared with the normal group, the majority of immune subpopulations trafficking to tumors were significantly increased with recruiting activities in the abnormal group (**Figure 5A**), including T cells, T helper 1 (Th1), Th17, Th22 cells, dendritic cells (DC), macrophages, nature killer (NK), and B cells. Meanwhile, CD4^+^T, Th2, regulatory T (Treg) cells, monocyte, neutrophil, and myeloid-derived suppressor cells (MDSCs) were decreased. Interestingly, alteration patterns of the abnormal group were highly similar to those that occurred in AIDs (27, 28). We subsequently analyzed immune activities against tumors (**Figure 5B**). Activities of cancer antigen release and immune cell infiltration into tumors were significantly enhanced, while cancer antigen presentation and cancer cell recognition by T cells were decreased in the abnormal group. No significant difference in CD8^+^T cell recruiting and activity of cancer cell killing was observed. These findings indicated that specific immune activities were enhanced in the abnormal group and resulted in inefficient antitumor activities. However, neither of the immune subpopulations and immune activities had significant differences among the three molecular subtypes of patients with multiple abnormal immunologic markers (**Figures 5C, D**).

## Discussion

Until now, there have been very few studies on DLBCL with AIDs, which all came to the same conclusion that this subgroup of patients is associated with inferior prognosis (3, 4) (**Table 2**). In this study, using integrated genomic and transcriptomic analyses, we not only revealed high-risk clinical characteristics and poor clinical outcome but also distinct molecular features, gene expression pattern, and microenvironment alternation of DLBCL with multiple abnormal immunologic markers. Among gene mutations, *CCND3* and *HIST1H1E* are the two genes with the highest frequency of mutation, the former one being mainly associated with cell cycle progression (12), and the latter with histone methylation (31). Analyses of oncogenic signaling pathways and biological processes demonstrated that cell cycle and immune response processes were significantly dysregulated in the abnormal group, further correlating the gene mutations with biological functions in DLBCL with multiple abnormal immunologic markers. Besides, DEL represented only 10%–15% of all enrolled patients, lower than epidemiological data (2) in both the abnormal and normal groups, probably due to the limited sample size of our study.

**Table 2:** Main results of references about AIDs and lymphoma.

Subjects	Proportion of AIDs	Clinical features	Prognosis	References
**612 DLBCL**	17.3%	Thyroid disease dominated followed by RA. The proportion of AIDs was significantly higher in females.	Patients with AIDs primarily mediated by B-cell responses had a worse OS.	Morth et al., 2019 (4)
**736 DLBCL, 703 FL, 302 MZL, 193 MCL, 297 HL, and 186 T-cell lymphoma**	12.2% in DLBCL	RA was the most common autoimmune condition and was the highest in MZL (7.6%), followed by DLBCL (7.2%).	Patients with AIDs primarily mediated by B-cell responses had an inferior EFS in MCL and HL.	Kleinstern et al., 2018 (29)
**435 B-NHL**	22.5% in DLBCL	Time to relapse for all B-NHL patients with AIDs was significantly shorter than patients without AIDs, specifically in patients with DLBCL.	A history of B-cell-mediated AIDs was associated with shorter PFS and OS.	Kleinstern et al., 2018 (30)
**1,771 DLBCL, 1,760 MM, 1,580 CLL, 936 MZL, and 787 FL**	6.3% in DLBCL	Significantly increased risks for DLBCL and MZL were found for those with rheumatological disorders; the site distribution of those with/without rheumatological conditions.	The 1- and 3-year OS rates of patients with three or more preceding rheumatology episodes were 59.5% and 46.6%, respectively, which were significantly poorer than those of patients without rheumatology episodes in DLBCL.	Kane et al., 2019 (3)

ECOG, Eastern Cooperative Oncology Group; LDH, lactate dehydrogenase; IPI, International Prognostic Index; DLBCL-NOS, diffuse large B-cell lymphoma, not otherwise specified; EBV^+^ DLBCL, Epstein–Barr virus-positive diffuse large B-cell lymphoma; PCDLBCL-LT, primary cutaneous diffuse large B-cell lymphoma, leg type; DEL, double expressor lymphoma; GCB, germinal center B-cell-like; ABC, activated B-cell-like; UN, unclassified; CR, complete response; PR, partial response; SD, stable disease; PD, progressive disease; AIDs, autoimmune diseases; RA, rheumatoid arthritis; EFS, event-free survival; OS, overall survival; FL, follicular lymphoma; MZL, marginal zone lymphoma; MCL, mantle cell lymphoma; HL, Hodgkin lymphoma; B-NHL, B-cell non-Hodgkin lymphoma; MM, multiple myeloma; CLL, chronic lymphocytic leukemia.

Primary BCR-mediated AIDs (RA, SLE, SS, etc.) are associated with the pathogenesis of DLBCL (29, 30). Chronic inflammation and antigen stimulation of AIDs may also contribute to lymphoma progression. It is well recognized that GC B cells alternate between proliferation and somatic hypermutation (SHM) in the dark zone and affinity-dependent selection in the light zone during maturation. Random SHM leads to the occurrence of mutations, resulting in self-antigen recognition and AID attack (32). Cyclin D3 (encoded by *CCND3*) controls the level of B-cell proliferation in the dark zone in a dose-dependent manner, essential for GC B-cell cloning and response to T follicular helper (Tfh) cells (33). Moreover, *CCND3* mutation promotes the acquisition of clonal lymphoproliferative phenotypes of B cells, which could potentially act as a pathogenic mechanism of DLBCL with AIDs.

Dysregulated BCR, Toll-like receptors (TLR), and cytokine signaling are also necessary to initiate spontaneous, autoimmune GC responses, resulting in loss of T-cell tolerance, epitope spreading, and GC-dependent systemic autoimmunity (34). During the increase of BCR affinity, elevated OxPhos promotes positive selection of B cells by tuning cell division in GCs (35). This is consistent with our findings on gene expression pattern that the OxPhos pathway is significantly activated in DLBCL with multiple abnormal immunologic markers. On the other hand, lymphoma cells could adapt to intrinsic oxidative stress by enhancing mitochondrial biogenesis, which is relevant to the acquisition of newly formed mitochondria transferred by mesenchymal stromal cells, leading to increased OxPhos, drug resistance, and lymphoma relapse (9, 10). According to the Consensus Cluster Classification, OxPhos-DLBCL is characterized by increased expression of proteasomal subunits and molecules that regulate mitochondrial membrane potential and apoptosis. This subset of DLBCL might thus be sensitive to proteasome blockade or inhibition of the BCL-2 family (11). However, the efficacy of proteasome and BCL-2 inhibitors warrants further investigations in clinical trials. In addition, OxPhos-related metabolic inhibitors could also become potential treatment options (36).

As for the tumor microenvironment, immune cell alterations play an important role in AID progression. Th1/Th2 and Th17/Treg ratios were increased in AIDs (27, 28). The Th1/Th2 ratio impacts the susceptibility of an individual to infections, allergy, and autoimmunity. Th1 cells are relevant to the pathogenesis of AID RA, multiple sclerosis, and Hashimoto thyroiditis, while Th2 cells to AID SLE (37). OxPhos is implicated in fate decision of Th17 and Treg cells by supporting early molecular events that are necessary for Th17 commitment (38). In our study, the state of antitumor immunity is also analyzed in DLBCL with the dysregulation of specific T-cell subsets, such as more Th1, Th22, and Th17 cells and fewer Th2 and regulatory Treg cells. These findings are consistent with changes of T-cell subsets in AIDs and provide a theoretical basis for immunoregulatory therapy in this subset of DLBCL.

To conclude, multiple abnormal immunologic markers may contribute to lymphoma progression. DLBCL with multiple immunologic marker abnormalities is featured by dysregulated cell cycle and immune response and activated OxPhos pathway, providing a clinical rationale of using mechanism-based targeted therapy in this subset of DLBCL.

## Data Availability Statement

The datasets presented in this study can be found in online repositories. The names of the repository/repositories and accession number(s) can be found below: https://www.biosino.org/node/review/detail/OEV000208?code=IXF5BJJE, https://www.biosino.org/node/review/detail/OEV000206?code=KO6DQQGF.

## Ethics Statement

The studies involving human participants were reviewed and approved by Shanghai Ruijin Hospital Ethics Board. Written informed consent to participate in this study was provided by the legal guardian/next of kin of the participants.

## Author Contributions

LS, DF, YC, YH, RS, PX, LW, and WZ conceptualized and designed the study. DF, YC, SW, and RS collected the data and prepared the biological samples. LS and DF analyzed the data. LS and WZ drafted the manuscript. PX, SC, LW, and WZ provided administrative, technical, and material support and supervised this study. PX, LW, and WZ acquired the funding. All authors contributed to the article and approved the submitted version.

## Funding

This work was supported by the National Natural Science Foundation of China, Grant/Award Numbers: 82130004, 81830007, 82070204, and 81670176; Clinical Research Plan of Shanghai Hospital Development Center, Grant/Award Number: SHDC2020CR1032B; Shanghai Municipal Education Commission Gaofeng Clinical Medicine, Grant/Award Numbers: 20152206 and 20152208; Multicenter Clinical Research Project by Shanghai JiaoTong University School of Medicine, Grant/Award Number: DLY201601; and Chang Jiang Scholars Program and Samuel Waxman Cancer Research Foundation.

## Conflict of Interest

The authors declare that the research was conducted in the absence of any commercial or financial relationships that could be construed as a potential conflict of interest.

## Publisher’s Note

All claims expressed in this article are solely those of the authors and do not necessarily represent those of their affiliated organizations, or those of the publisher, the editors and the reviewers. Any product that may be evaluated in this article, or claim that may be made by its manufacturer, is not guaranteed or endorsed by the publisher.

## Supplementary Material

Supplementary Figure S1 Dysregulated signaling pathways of DLBCL with CCND3 or *IRF4* mutation. **(A)** Dysregulated signaling pathways of DLBCL with (*n* = 8) or without (*n* = 105) *CCND3* mutation analyzed by GSEA *via* KEGG database. **(B)** Dysregulated signaling pathways of DLBCL with (*n* = 8) or without (*n* = 105) *IRF4* mutation analyzed by GSEA *via* GO database. Color of points indicates −log (P value) of dysregulated pathways of two groups. Size of points indicates number of genes included in each gene set.
