# Supplementary figures and images for: Integrated Genomic and Transcriptomic Analyses of Diffuse Large B-Cell Lymphoma With Multiple Abnormal Immunologic Markers
Source: Front Oncol. 2022 Feb 14;12:790720. doi: 10.3389/fonc.2022.790720 (PMC8882913; doi:10.3389/fonc.2022.790720)

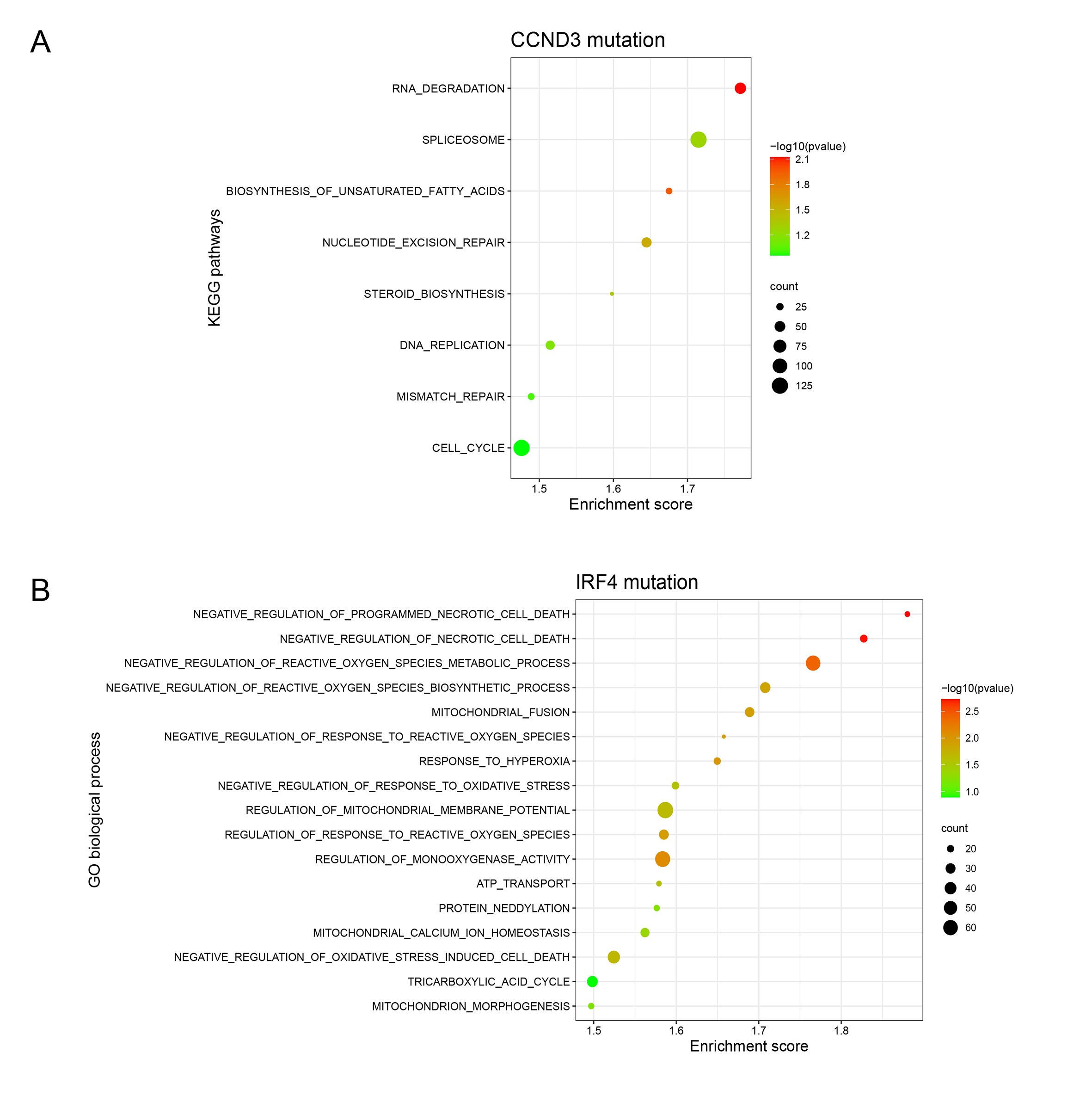

Supplement: Supplementary Figure S1 — Dysregulated signaling pathways of DLBCL with CCND3 or IRF4 mutation. (A) Dysregulated signaling pathways of DLBCL with (n = 8) or without (n = 105) CCND3 mutation analyzed by GSEA via KEGG database. (B) Dysregulated signaling pathways of DLBCL with (n = 8) or without (n = 105) IRF4 mutation analyzed by GSEA via GO database. Color of points indicates −log (P value) of dysregulated pathways of two groups. Size of points indicates number of genes included in each gene set. [file Image_1.tif]
